# *Candida glabrata* infection following total hip arthroplasty: A case report

**DOI:** 10.3892/etm.2013.1420

**Published:** 2013-11-21

**Authors:** YUN ZHU, CHEN YUE, ZEYU HUANG, FUXING PEI

**Affiliations:** Department of Orthopaedics, West China Hospital, Sichuan University, Chengdu, Sichuan 610041, P.R. China

**Keywords:** candida glabrata, infection, total hip arthroplasty

## Abstract

*Candida glabrata* infection following total hip arthroplasty is rare and, due to the insufficiency of standardized clinical and evidence-based guidelines, there is no appropriate therapeutic schedule. The present study reports the case of a 44-year-old patient with *Candida glabrata* infection following a total hip arthroplasty. The patient was successfully treated by administration of intravenous and oral voriconazole without removal of the prosthesis. This case illustrates the significance of postoperative follow-up, clinician experience and the choice of the correct antifungal agent. In this case, we found in the early stage of *Candida glabrata* infection, we were able to control the infection without surgery through thorough irrigation. This reduces patient suffering and economic burden.

## Introduction

*Candida glabrata* infection following total hip arthroplasty is a potentially devastating complication. If identified late, removal of the prosthesis and a course of appropriate systemic antifungal therapy are required. In knee or hip arthropalsty surgery, there are comparatively higher risk of infection than with smaller joints, due to the longer operation time and the low blood flow. Only when the symptom period for the patient was a short-time span, it is reasonable to leave the prosthesis in the patient and use debridement to remove the infection (1). The present study reports a case of *Candida glabrata* infection that arose under the incision following a total hip arthroplasty, in which removal of the prosthesis was avoided. To the best of our knowledge, this is the first report of a case of *Candida glabrata* infection around an incision in which the prosthesis has been preserved.

## Case report

**Figure 1:**
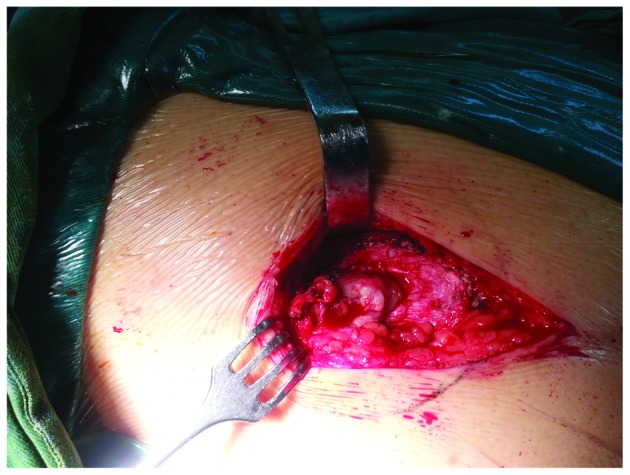
Cyst resembled tuberculosis intraoperatively.

**Figure 2:**
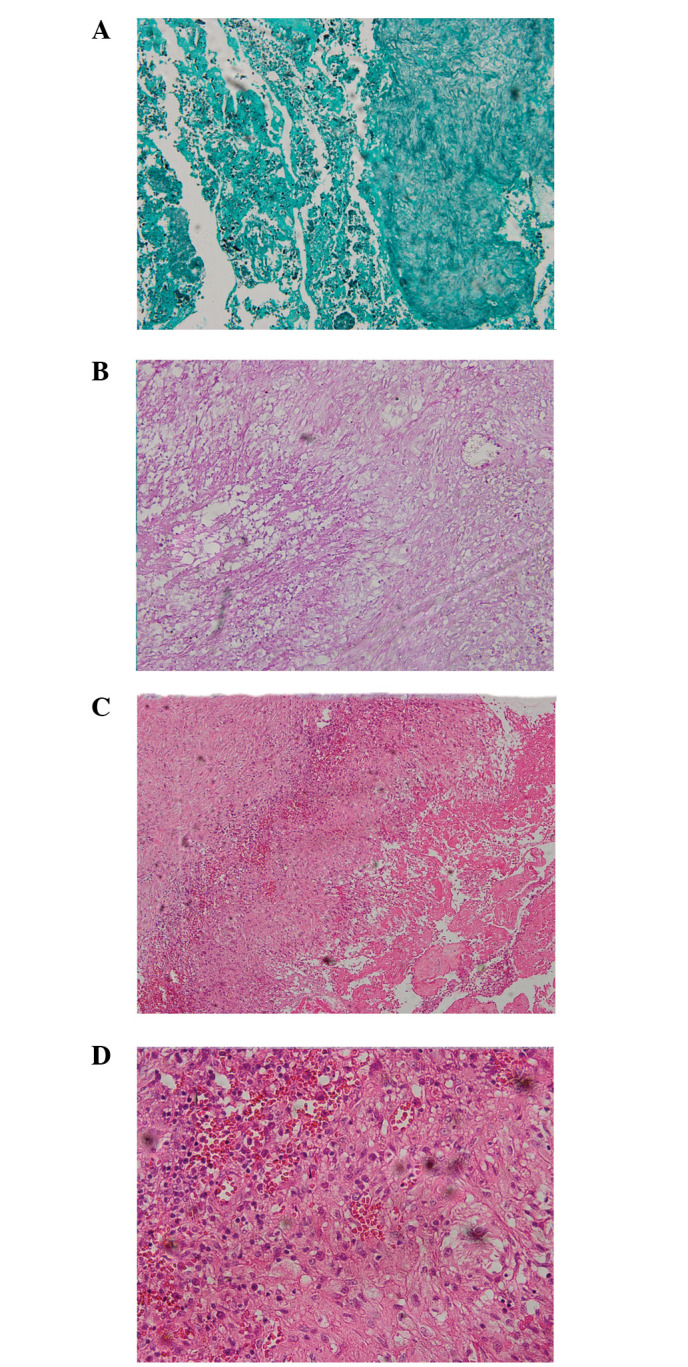
Staining showed positive granular material and small spherical objects. H&E staining showed chronic inflammation and granulation tissue. (A) Methenamine silver staining; (B) PAS staining; (C) H&E staining (magnification, ×10); and (D) H&E staining (magnification, ×40).

**Figure 3:**
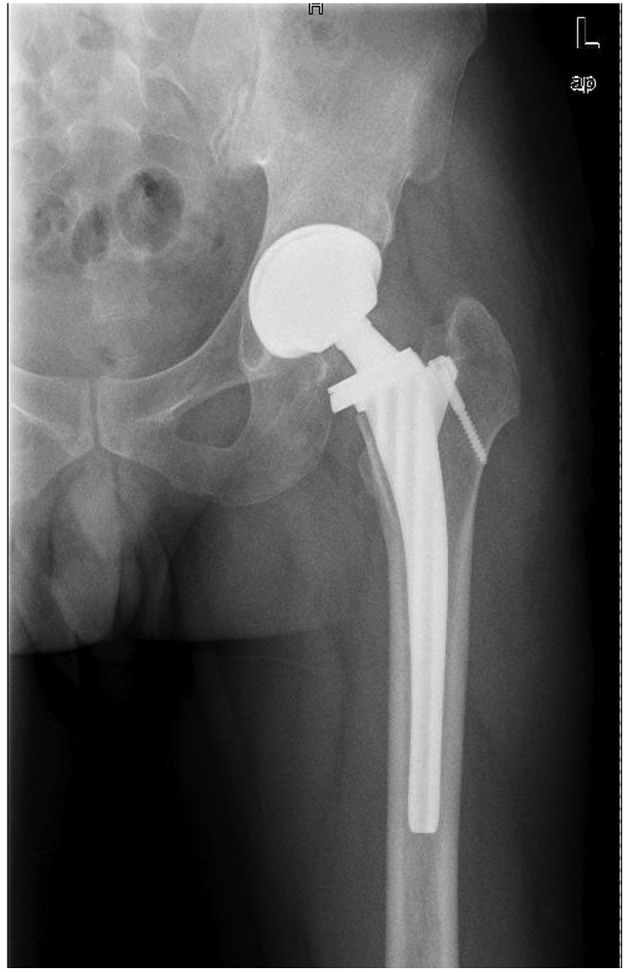
Imageological examination revealed no signs of prosthetic-loosening or infection.

A 44-year-old male patient underwent a bilateral total hip arthroplasty due to osteonecrosis of the femoral head in a two-stage surgery. During the first stage, the patient underwent the left total hip arthroplasty. Prior to receiving the right total hip arthroplasty, light subcutaneous swelling and redness was identified at the distal incision on the left side. The patient exhibited no symptoms of prosthetic loosening or infection, such as fever, chills, start-up pain or pain at rest. A probable infection around the incision was suspected. A one-month course of vancomycin was administered intravenously to the patient. Following this, the swelling had disappeared and the patient received the right total hip arthroplasty. However, 2 months later, the patient presented to the West China Hospital (Chengdu, China) with a 20 day-history of subcutaneous swelling of the left side distal incision. The patient continued to exhibit no common symptoms of joint infection and the active range of motion was normal. Other clinical findings included elevated inflammation markers, including a C-reactive protein (CRP) level of 22 mg/l (normal value, <5 mg/l) and an erythrocyte sedimentation rate (ESR) of 44 mm/h (normal value, <21 mm/h). Ultrasound confirmed the presence of a cyst that was not connected with the articular cavity. The patient was diagnosed with a superficial infection and the debridement of soft tissues was performed. Intraoperatively, it was noted that the contents of the cyst resembled tuberculosis (Fig. 1). Postoperatively, the patient was administered isoniazid and rifapentine while awaiting a microbiology report. The results of the report showed that *Candida glabrata* was present. The pathological section of the specimen revealed fungal infection and chronic inflammation (Fig. 2). Methenamine silver staining showed black Candida, PAS staining showed purple Candida and H&E staining showed edema tissue and inflammatory cell infiltration. The *Candida glabrata* isolate showed susceptibility to itraconazole [minimum inhibitory concentration (MIC), 2 μg/ml], amphotericin B (MIC, 0.5 μg/ml), 5-fluorocytosine (MIC, 4 μg/ml), fluconazole (MIC, 8 μg/ml) and voriconazole (MIC, 1 μg/ml). The patient was initially administered intravenous amphotericin B in escalating doses. When the dosage of amphotericin B was increased up to 1 mg/kg per day, the patient refused to continue receiving amphotericin B due to severe gastrointestinal reactions. Consequently, the patient was switched to voriconazole. The patient tolerated the 6-week course of antifungal treatment without any adverse events, and the CRP level and ESR returned to normal. The redness and swelling at the distal operative site disappeared. Aspiration of the hip was also negative. At the 3 month follow-up the patient did not exhibit swelling and the range of motion of the left hip was normal. Imageological examination showed no signs of prosthetic-loosening or infection (Fig. 3).

## Discussion

*Candida glabrata* infection following total hip arthroplasty is a potentially devastating complication. Moreover, in the absence of standardized clinical and evidence-based guidelines, it is difficult to manage. *Candida glabrata* has been historically considered as a relatively nonpathogenic saprophyte and rarely causes serious infection in humans. However, with widespread use of immunosuppressive drugs, broad-spectrum antibiotics and azole antifungals, *Candida glabrata* is now more frequently isolated from clinical specimens (2). There are three possible etiologies of *Candida glabrata* infection. These include direct seeding via trauma, iatrogenic causes (surgery) and hematogenous spread (3). In the present case, the patient had a medical history of prolonged antibiotic treatment. However, another potential risk factor is that the cyst was located in the muscle layer. It is assumed there was a large amount of dead-space in the muscle layer during the surgery. Hematoma formation may occur within this dead-space and may disrupt blood supply to the surrounding tissue, thus preventing antibiotic entry (4).

Routine treatment usually includes the surgical removal of all bioprosthetic components. Early-onset infections may be eradicated by debridement and a long course of parenteral antibiotics. Antibiotic therapy is based on the definitive microbiological diagnosis and the sensitivity to the antibiotics. Generally, 6 weeks of parenteral antibiotics are recommended for prosthetic joint infections (5). Postoperatively, the patient did not exhibit symptoms of infection, including fever, vomiting and groin pain. The radiograph of the bilateral hip did not reveal the presence of any aseptic loosening of a component of the prosthesis. Ultrasound confirmed that the cyst was not connected with the articular cavity. The active range of motion of the left hip was invariably normal postoperatively. Repeated aspiration of the left hip was negative. Consequently, the decision was made to retain the prosthesis.

The appropriate course of antibiotic treatment was selected, based on the sensitivity of the infection to specific antibiotic agents. The protocols for the treatment of infections associated with hip arthroplasty, which include 6 weeks of parenteral treatment, have been demonstrated previously (6–8). Due to the severe gastrointestinal reactions of the patient to amphotericin B, voriconazole was administered instead. This was the antifungal drug to which the infection had the second highest susceptibility in the present case. Following 6 weeks of voriconazole treatment, normalization of CRP and ESR was achieved.

*Candida glabrata* infection following total hip arthroplasty is extremely rare. This infection is generally asymptomatic or gives rise to mild signs of infection in the early stages. If identified late, diffusion of the infection may result in irreversible deformity and pain with severe osteoarticular destruction (9). Thus, early diagnosis and treatment are important in the management of *Candida glabrata*. If there are minimal signs of infection following arthroplasty, close co-operation between the clinician and laboratory are required in order to identify the infectious agent.

The present case illustrates the significance of postoperative follow-up and the experience of the clinician. If a patient presents abnormal symptoms without signs of common infection following hip arthroplasty, the possibility of a fungal infection should be considered.
